# Recommendations for prioritization, treatment, and triage of breast cancer patients during the COVID-19 pandemic. the COVID-19 pandemic breast cancer consortium

**DOI:** 10.1007/s10549-020-05644-z

**Published:** 2020-04-24

**Authors:** Jill R. Dietz, Meena S. Moran, Steven J. Isakoff, Scott H. Kurtzman, Shawna C. Willey, Harold J. Burstein, Richard J. Bleicher, Janice A. Lyons, Terry Sarantou, Paul L. Baron, Randy E. Stevens, Susan K. Boolbol, Benjamin O. Anderson, Lawrence N. Shulman, William J. Gradishar, Debra L. Monticciolo, Donna M. Plecha, Heidi Nelson, Katharine A. Yao

**Affiliations:** 1 National Accreditation Program for Breast Centers, Chicago, IL USA; 2 American Society of Breast Surgeons, Columbia, MD USA; 3 National Comprehensive Cancer Network, Plymouth Meeting, PA USA; 4 Commission On Cancer, Chicago, IL USA; 5 American College of Radiology, Reston, VA USA; 6 University Hospital Cleveland Medical Center, Cleveland, OH USA; 7 Yale Medicine, New Haven, CT USA; 8 Massachusetts General Hospital Cancer Center, Boston, MA USA; 9 Waterbury Hospital, Waterbury, CT USA; 10 Inova Schar Cancer Institute, Fairfax, VA USA; 11 Dana Farber Cancer Institute, Boston, MA USA; 12 Fox Chase Cancer Center, Philadelphia, PA USA; 13 Carolinas Medical Center, Charlotte, NC USA; 14 Montefiore Medical Center, Bronx, NY USA; 15 White Plains Hospital, White Plains, NY USA; 16 Nuvance Hospital, Poughkeepsie, NY USA; 17 University of Washington, Seattle, WA USA; 18 University of Pennsylvania, Philadelphia, PA USA; 19 Northwestern Medicine, Chicago, IL USA; 20 Baylor Scott & White Healthcare-Central Texas, Temple, TX USA; 21 NorthShore University HealthSystem, Evanston, IL USA

## Abstract

The COVID-19 pandemic presents clinicians a unique set of challenges in managing breast cancer (BC) patients. As hospital resources and staff become more limited during the COVID-19 pandemic, it becomes critically important to define which BC patients require more urgent care and which patients can wait for treatment until the pandemic is over. In this Special Communication, we use expert opinion of representatives from multiple cancer care organizations to categorize BC patients into priority levels (A, B, C) for urgency of care across all specialties. Additionally, we provide treatment recommendations for each of these patient scenarios. Priority A patients have conditions that are immediately life threatening or symptomatic requiring urgent treatment. Priority B patients have conditions that do not require immediate treatment but should start treatment before the pandemic is over. Priority C patients have conditions that can be safely deferred until the pandemic is over. The implementation of these recommendations for patient triage, which are based on the highest level available evidence, must be adapted to current availability of hospital resources and severity of the COVID-19 pandemic in each region of the country. Additionally, the risk of disease progression and worse outcomes for patients need to be weighed against the risk of patient and staff exposure to SARS CoV-2 (virus associated with the COVID-19 pandemic). Physicians should use these recommendations to prioritize care for their BC patients and adapt treatment recommendations to the local context at their hospital.

## Introduction

The COVID-19 pandemic poses unprecedented challenges for patients, clinicians, and healthcare systems. Across every facet of medicine, clinicians are responding to the pandemic by modifying patient care to minimize exposure risk and preserve resources, and the management of patients with cancer poses unique challenges [1]. To provide preliminary guidance on the prioritization and treatment of breast cancer (BC) during this severe acute respiratory syndrome coronavirus 2 (SARS-CoV-2) outbreak, we assembled representatives from the American Society of Breast Surgeons (ASBrS), the National Accreditation Program for Breast Centers (NAPBC), the National Comprehensive Care Network (NCCN), the Commission on Cancer (CoC) and the American College of Radiology (ACR) to formulate an Expert Opinion. The objective of this Special Communication is to prioritize patient scenarios by urgency of treatment by specialty and to make treatment recommendations based on these priorities within each specialty. Given the rapidly evolving nature of the COVID-19 pandemic, time constraints prohibited a formal consensus statement.

These recommendations relate specifically to BC patients not suspected to have COVID-19-related illness. We acknowledge that there are limited prospective experiences to guide these recommendations. Furthermore, these recommendations are driven by the common goal to preserve hospital resources for virus-inflicted patients by deferring BC treatments without significantly compromising long-term outcomes for individual BC patients. The demands that the COVID-19 pandemic will place on healthcare institutions remain unpredictable and will have geographical variability. Therefore, the risks of disease progression and compromised BC-specific outcomes need to be weighed against viral exposure to patients and staff, taking into consideration each individual’s comorbidities and age to predict risk of mortality from COVID-19. Lastly, these are recommendations and are not intended to supersede individual physician judgment or institutional policies and guidelines.

## Methods

After extensive multidisciplinary teleconference discussions and literature review, a “Priority” classification for BC patients was developed across the disciplines. Priority categories were defined based on the severity of an individual patient’s condition (including patient comorbidities) and potential efficacy of treatments [2].

### Priority A category

Priority A patients have a condition that is immediately life threatening, clinically unstable, or completely intolerable and for whom even a short delay would significantly alter the patient’s prognosis. Assuming efficacious treatment, these patients are given top priority even if resources become scarce, requiring urgent treatment for preservation of life or control of progressing disease or symptomatic relief.

### Priority B category

Patients in the Priority B category are patients who do not have immediately life-threatening conditions but for whom treatment or services should not be indefinitely delayed until the end of the pandemic. Most BC patients will fall under Priority B. If conditions in a geographic location only allow for Priority A patients to receive treatment, then treatment for Priority B patients can be delayed for a defined period of time during the pandemic. A short delay (e.g. 6–12 weeks) would not impact overall outcome for these patients. Longer delays could impact outcomes in some Priority B patients and triage may become necessary to justify which patients should undergo treatment versus further delay. Patients within the Priority B category will be sub-stratified as B1 (higher priority), B2 (mid-level priority), and B3 (lower priority) as defined by each BC subspecialty.

### Priority C category:

Patients in Priority C category are patients for whom certain treatment or services can be indefinitely deferred until the pandemic is over without adversely impacting outcomes.

## Results

Priority categories and treatment recommendations by specialty are listed below.

## Outpatient visits

During the pandemic, the majority of encounters should be conducted remotely via telemedicine. Decisions to conduct in-person visits must carefully weigh the risk of viral transmission to patients and healthcare providers with the need for an in-person evaluation. Priority A includes, for example, clinically unstable postoperative patients and those with potential medical oncologic emergencies (e.g. febrile neutropenia, intractable pain) who need to be assessed in-person. Priority B patients should be evaluated by at least one member of the multidisciplinary team in-person or remotely depending on need. These include newly diagnosed BC patients; established patients with new problems (breast infection, palpable findings, and significant symptoms from therapy); patients on active IV chemotherapy; patients completing neoadjuvant therapy preparing for surgery; routine postoperative patients; and patients being evaluated and planned for radiation therapy. Priority C patients are those presenting for routine follow-up for benign or malignant conditions (including those on oral adjuvant agents and those not on active treatment), survivorship visits, or high-risk screening and can be seen remotely or delayed until the postpandemic period. Increased precautions should be taken surrounding in-person visits/treatments for patients with comorbidities and a high risk of COVID-19 complications.

## Breast focused imaging

Few scenarios are designated Priority A for breast imaging, with the exception of imaging for urgent situations such as a severe breast abscess formation or for evaluation of a serious postoperative complication..

Breast imaging Priority B includes diagnostic imaging for an abnormal mammogram or for suspicious breast symptoms, biopsies for BI-RADS 4 or 5 lesions, and breast MRI for extent of disease evaluation or pre-chemotherapy assessment. Biopsies for lower suspicion lesions (BI-RADS 4a) may be postponed or biopsied. BI-RADS category 3 patients returning for short-term follow-up diagnostic mammogram and/or ultrasound and routine breast examination should be postponed until the COVID-19 pandemic is over and would be Priority C. All screening examinations including mammography, ultrasound, and MRI should be placed in Priority C and suspended until the post-COVID-19 period. BRCA mutation carriers under the age of 40 may be considered for screening if delays of more than 6 months are expected [3, 4].

## Surgical oncology

Table 1 lists patient scenarios into Priority categories for urgency of surgical care. The need to minimize use of operating room resources requires selectively deferring surgery and triaging patients for use of an initial alternative therapy whenever possible. However, level II evidence demonstrates that preoperative delays may impact BC outcomes [5, 6].

**Table 1:** Priority categories for surgical oncology

Priority	Patient description	COVID-19 treatment considerations
Priority A		
A	Breast abscess in a septic patient	Operative drainage if unable to be drained at the bedside
A	Expanding hematoma in a hemodynamically unstable patient	Operative evacuation and control of bleeding
Priority B		
B1	Ischemic autologous tissue flap	Revascularize or remove flap
B1	Revision of a full thickness ischemic mastectomy flap with exposed prosthesis	Debride and remove expander/implant
B1	Patients who have completed neoadjuvant chemotherapy for Inflammatory BC	Operate as soon as possible depending on institutional resources*
B1	TNBC and HER2 + patients	Neoadjuvant chemotherapy or HER2 targeted therapy. In some cases, institutions may decide to proceed with surgery first versus neoadjuvant therapy. These decisions will depend on institutional resources and patient factors.*
B2	Neoadjuvant: -finishing treatment -progressing on treatment	Operate if feasible depending on resources or extend/change neoadjuvant therapy*
B3	Clinical Stage T2 or N1 ER + / HER2 – tumors	Consider hormonal treatment, delay operation
B3	Discordant biopsies likely to be malignant	Perform excisional biopsy when conditions allow
B3	Malignant or suspected local recurrence	Begin with staging when feasible. Perform excision when conditions allow if there is no distant disease
Priority C		
C1	ER–DCIS	Delay operation until after COVID-19 unless there is a high risk of invasive cancer (Move to B3)
C1	Positive margin(s) for invasive cancer	Delay re-excision until after COVID-19
C1	Clinical Stage T1N0 ER + / HER2—cancers	Hormonal treatment; delay operation until after COVID-19
C1	BC patients requiring additional axillary surgery	Delay operation until after COVID-19
C2	ER + DCIS	Hormonal treatment; delay operation until after COVID-19
C2	High-risk lesions	Delay operation until after COVID-19
C2	Reconstruction for previously completed mastectomy	Delay operation until after COVID-19
C3	Excision of benign lesions-fibroadenomas, nodules, papillomas, etc	Delay operation until after COVID-19
C3	Discordant biopsies likely to be benign	Delay operation until after COVID-19
C3	Prophylactic surgery-for cancer and noncancer	Delay operation until after COVID-19

^*^Breast conservation is preferred provided that radiation oncology services are available, and the risk of multiple visits or deferred radiation is acceptable. If no ventilator is available or risk of viral exposure is high, breast conserving surgery could be performed under local with sedation. Reconstruction should be limited to tissue expander or implant placement if necessary depending on institutional resources. Autologous reconstruction should be deferred

*BC* breast cancer, *TNBC* triple negative breast cancer, *ER* estrogen receptor, *HER2* human epidermal growth factor receptor 2, *DCIS* ductal carcinoma in situ

Invasive BC patients should be triaged with multidisciplinary input and assessment of patient’s risks and comorbidities to potentially receive neoadjuvant therapies during the pandemic. While neoadjuvant chemotherapy confers risks of immunosuppression and uses personal protective equipment (PPE), high-risk breast cancers would fall in Priority B because upfront surgery is not required when systemic treatment is initiated. Current standards for triple negative breast cancer (TNBC) and human epidermal growth factor 2-overexpressing (HER2 +) BC already include neoadjuvant therapy, which has very high rates of clinical and pathological tumor response affording durable tumor control prior to deferred surgery [7, 8]

Patients completing neoadjuvant chemotherapy are categorized as Priority B1. Delays of surgery up to 8 weeks postchemotherapy do not adversely affect BC outcomes [9]. Breast imaging cannot be used as a surrogate to assess pathologic response because false negative rates vary between 17.8 and 50% [10–13] In the event that resources do not allow for surgery, additional non-surgical therapy should be considered (see Medical Oncology section).

Patients with hormone receptor-positive BC are Priority B3 or C because neoadjuvant endocrine therapy allows for deferment of definitive surgery. Studies evaluating tamoxifen with/without surgery demonstrate no difference in survival within the first three years suggesting that short-term deferment of surgery with endocrine therapy should not adversely impact BC-specific survival [14–16]

Patients eligible for breast conservation should be discouraged from elective mastectomy depending on local institutional resources. For patients requiring mastectomy, immediate reconstruction with implant or tissue expanders can be performed only if hospital resources permit. Autologous reconstruction should be deferred [17]

Discordant biopsies are uncommon, but when they occur, establishing the presence of malignancy is required [18]. These patients would be categorized in Priority B or C depending on level of suspicion.

For newly diagnosed, recurrent BC, staging evaluation is preferred but may be unavailable. Surgery is typically indicated only in the absence of metastatic disease. Treatment will depend on resource availability (see Medical Oncology section).

Re-operation for margins or axillary staging is Priority C when there is a low likelihood of residual disease [19]. Patients with estrogen receptor-positive (ER +) ductal carcinoma in situ (DCIS) and low volume ER- DCIS are Priority C1 whereas patients at high-risk for occult invasion are Priority B3. Non-operative trials, however, are limited to low-risk DCIS [20–22].

Practitioners caring for BC see many benign conditions. If a malignant lesion is unlikely, diagnostic procedures should be postponed. It is advisable to follow-up with patients whose treatment is being altered or postponed. The enormity of changes from the COVID-19 pandemic itself is anxiety-provoking among patients and practitioners. Patient psychological well-being needs to be considered and often can be addressed with telemedicine/phone visits. While shared decision-making is ideal, in the context of the pandemic difficult choices must be made.

## Medical oncology

Table 2 lists patient scenarios into Priority categories for urgency of either hormonal, chemotherapy and/or targeted therapy. The medical oncology goals are to minimize patient interactions with healthcare centers, maintain patient safety, and conserve resources while providing effective care. All specialty and institutional goals and patient factors should be considered when formulating a treatment plan. Priority A patients are those with oncologic emergencies requiring immediate treatment (e.g. febrile neutropenia, intractable pain). Priority B patients require systemic care but are candidates for modified therapeutic approaches to achieve the goals above; the urgency and therapeutic options are stratified into higher-to-lower priorities (B1-B3). Priority C patients can delay interventions for many months without adverse impact on survival or quality of life.

**Table 2:** Priority categories for medical oncology

Priority	Patient description	COVID-19 treatment considerations
Priority A		
A	Patients with oncologic emergencies (e.g. febrile neutropenia, hypercalcemia, intolerable pain, symptomatic pleural effusions or brain metastases, etc.)	Initiate necessary management
Priority B		
B1	Patients with inflammatory BC	Neoadjuvant chemotherapy
B1	Patients with TNBC or HER2 + BC	Neo/adjuvant chemotherapy (Neoadjuvant for ≥ T2 or N1)
B1	Patients with mBC for whom therapy is likely to improve outcomes	Initiate chemotherapy, endocrine, or targeted therapy
B1	Patients who already started neo/adjuvant chemotherapy	Continue therapy until complete (if neoadjuvant and responding, can extend treatment if necessary to defer surgery further)
B1	Patients progressing on neoadjuvant therapy	Refer to surgery or change systemic therapy
B1	Patients on oral adjuvant endocrine therapy	Continue therapy
B1	Premenopausal patients with ER + BC receiving LHRH agonists (adjuvant or metastatic)	- If on aromatase inhibitor, continue LHRH agonist and consider long acting 3 month dosing or home administration - If on tamoxifen, consider deferring LHRH agonist
B1	Patients with clinical anatomic Stage 1 or 2 ER + /HER2- BCs	Neoadjuvant endocrine therapy for 6 to 12 months to defer surgery (may consider gene expression assay on core biopsy)
B2	Patients receiving treatment for Stage 1 HER2 + breast	Ado-trastuzumab emtansine may be substituted for paclitaxel/ trastuzumab
B3	Patients with ER + DCIS	Consider neoadjuvant endocrine therapy to defer surgery
B3	Patients with mBC for whom therapy is unlikely to improve outcomes	Consider deferring chemotherapy, endocrine, or targeted therapy
B3	Patients with HER2 + mBC beyond 2 years of maintenance antibody therapy (trastuzumab, pertuzumab) with minimal disease burden	Consider stopping antibody therapy with monitoring for progression every 3–6 months
B3	Patients with HER2 + BC receiving adjuvant antibody treatment	Consider curtailing antibody treatment after 7 months instead of 12 months
Priority C		
C	Patients receiving zoledronic acid, denosumab	Discontinue bone antiresorptive therapy unless for hypercalcemia
C	Patients with stable mBC	Interval for routine follow-up restaging studies can be delayed
C	Patients with lower risk imaging findings needing follow-up (e.g., small pulmonary nodules)	Interval follow-up can be delayed
C	Patients who are candidates for prevention measures (e.g. family history, LCIS or ADH, BRCA1/2 +)	Consider endocrine therapy (as appropriate), delay surgery and screening imaging
C	Patients in long-term follow-up for early BC	Defer routine in-person visit
C	Patients on aromatase inhibitors	Defer bone density testing (baseline and follow-up)

*BC* breast cancer, *TNBC* triple negative breast cancer, *mBC* metastatic BC, *LHRH* luteinizing hormone releasing hormone, *ER* estrogen receptor, *HER2* human epidermal growth factor receptor 2, *DCIS* ductal carcinoma in situ, *LCIS* lobular carcinoma in situ, *ADH* atypical ductal hyperplasia

### Invasive BC—early stage

For newly diagnosed BC patients, multidisciplinary plans can be revised to protect patients and spare healthcare services (Priority B). Depending on local circumstances, surgery, systemic therapy, and radiation therapy (RT) sequencing may be altered to ensure patient safety and healthcare system needs. Neoadjuvant treatment is well established for all BC subtypes and enables delayed surgery. If necessary, RT can be given before adjuvant chemotherapy (especially for ER + tumors) without affecting long-term outcomes [23].

Patients with ER + , HER2- tumors can defer surgery and receive neoadjuvant endocrine therapy for 6 to 12 months without clinical compromise (Priority B1) [24, 25]. Patients should be assessed periodically to confirm the absence of tumor progression. Patients with Stage 1 or limited Stage 2 disease (including those with N1 nodal involvement), and those with low-intermediate grade tumors, lobular BCs, low-risk genomic assays (especially the recurrence score, which may be sent from a core biopsy [26]), or “luminal A” signatures, do not benefit substantially from neoadjuvant or adjuvant chemotherapy [27, 28]. These patients may receive endocrine therapy alone.

TNBC patients should receive standard chemotherapy approaches (Priority B1), and all BC subtypes currently receiving neoadjuvant or adjuvant treatment should complete standard regimens already underway. Abbreviated schedules or dose modified regimens may be considered. Single-agent sequential therapy may reduce treatment complications without compromising efficacy [29].

Patients with Stage 1 or 2, HER2 + BCs may consider ado-trastuzumab emtansine (± pertuzumab) with comparable efficacy to chemotherapy/trastuzumab-based regimens in either neoadjuvant or adjuvant settings (Priority B2) to minimize neutropenia, visits, and steroid-use [30, 31]. Adjuvant trastuzumab-based therapy may be shortened from 12 to 6 months without affecting outcomes in selected patients (Priority B3) [32, 33].

### Invasive BC—advanced stage

Patients with advanced (metastatic) BC have many treatment options and typically receive multiple lines of therapy which are rarely dependent on specific treatment sequencing. Dose and schedule adjustments of systemic treatments are reasonable to reduce clinic visits, bloodwork, and development of significant side effects. Patients without signs or symptoms of tumor progression may defer routine restaging scans. When the likely benefit of additional palliative chemotherapy is very small, patients may find the risks of treatment outweigh the possible gains in outcome.

Trastuzumab, pertuzumab, and related antibody–drug conjugates for HER2 + tumors may be given at less frequent dosing intervals, as necessary. Patients with HER2 + BC with > 2 years duration of tumor control and minimal disease burden with trastuzumab-based regimens may consider interrupting maintenance therapy [34].

The use of oral targeted agents (CDK4/6, mTOR, and PIK3CA inhibitors) in ER + , metastatic BC must be weighed against the increased risk of adverse events. Dose reductions can minimize treatment-related toxicities. CDK4/6 inhibitors as first-/second-line treatment offer clinical advantage, but may be delayed if the likelihood of tumor control is high with endocrine therapy alone (first line, no prior endocrine treatment, no visceral disease) [35]. Dose reduction of palbociclib does not diminish efficacy [36, 37].

### High-risk lesions and pre-invasive BC

High-risk lesions such as atypical hyperplasia and lobular carcinoma in situ express ER and are effectively treated with either tamoxifen or aromatase inhibitors [38, 39]. ER + DCIS can be treated with preoperative endocrine therapy for 6 months (Priority C) [40]. For management of ER- disease see Surgical Oncology section.

### Supportive care and additional considerations

Endocrine treatments (tamoxifen, aromatase inhibitors, luteinizing hormone releasing hormone (LHRH) agonist) are safe and can be continued thru the COVID-19 pandemic (Table 3). LHRH agonists may be given every 3 months, and home administration is an option [41]. Patients receiving chemotherapy should receive appropriate supportive care to reduce side effects; in particular, granulocyte colony-stimulating factor (G-CSF) should be used to minimize neutropenia, and can be considered for regimens with < 20% chance of febrile neutropenia not usually offered G-CSF. Interventions that alleviate severe symptoms should remain a high priority. Bone modifying treatments (intravenous bisphosphonates or denosumab) can be deferred in patients without hypercalcemia, on adjuvant therapy, or on long-standing courses of therapy.

**Table 3:** Additional considerations for priority categories for medical oncology

Agent	Dosing and scheduling considerations
Chemotherapy	Chemotherapy schedules may be modified to reduce clinic visits (using 2- or 3-week dosing, e.g.) or to reduce infection risk (using weekly dosing) for selected agents when appropriate
	For low-risk febrile neutropenia, outpatient regimens may be used
	Selected patients (particularly with ER + disease), can consider radiation before chemotherapy if this facilitates patient safety
Targeted therapy	The addition of oral targeted agents (CDK 4/6, mTOR, or PIK3CA inhibitors) to endocrine therapy may be delayed in first-line treatment, or in situations where endocrine therapy alone is providing or is likely to provide effective tumor control
	Cardiac monitoring (Echo, nuclear) during HER2 antibody therapy can be delayed or discontinued if clinically stable
	Consider reduced dose of oral targeted agents to optimize tolerability and minimize treatment-related toxicities
	Trastuzumab and pertuzumab for metastatic HER2 + BC may reasonably be administered at longer intervals (e.g. 4 weeks)
Endocrine therapy	Oral endocrine agents (e.g. tamoxifen, aromatase inhibitors) are not immunosuppressive and can be safely continued
	Fulvestrant is not immunosuppressive but requires monthly clinical administration
	Aromatase inhibitors are preferred over tamoxifen for neoadjuvant endocrine therapy (and LHRH agonists should be used for premenopausal women)
Supportive care	Extend venous access device (port) flush to 12 weeks or longer
	Consider peripheral venous access for IV chemotherapy if patient has sufficient veins and no existing port if institutional policies permit
	Administer G-CSF growth factor support to minimize neutropenia
	Limit dexamethasone when possible to reduce immunosuppression

*ER* estrogen receptor, *LHRH* luteinizing hormone releasing hormone, *HER2* human epidermal growth factor receptor 2, *IV* intravenous, *G-CSF* granulocyte colony-stimulating factor

## Radiation oncology

Radiation therapy (RT) plays an integral role in the treatment of many BCs. Patient-related factors (age, comorbidities) contributing to infection risk must be carefully weighed against the risk of worsened BC outcomes if delaying the anticipated local–regional and potential survival benefits of RT. Priority A includes patients presenting with symptomatic disease in whom short palliative RT regimens should be utilized [42–44]. Patients clinically progressing on neoadjuvant therapy should be considered for a longer definitive preoperative hypofractionated (HF) regimen to reduce risk of continued progression if surgically unresectable or resource constraints prohibit timely surgery [45, 46].

The majority of RT referrals will likely be Priority B requiring triage/deferment. Limited published data on RT delays in the definitive setting are inconsistent. Locally advanced or inflammatory patients may have worse outcomes after neoadjuvant chemotherapy if RT is delayed > 8 weeks (Priority B1) [47]. In contrast, delaying RT 20 weeks in early-stage, ER + patients after BCS demonstrates no difference in outcomes compared to 4–8 weeks (Priority B3) [48]. Since Priority B sub-stratifies by clinical-pathologic recurrence risk, B1 patients should be given priority over B2 or B3; hypofractionated (HF) regimens should be strongly considered whenever possible. Long-term outcomes with HF-RT utilizing 42.5 Gy/16 or 40 Gy/15 fractions demonstrate safety and efficacy similar to conventional fractionation which will reduce patient/staff exposure [45, 46]. Though regional-nodal and postmastectomy patients were under-represented in these trials, historic [49] and emerging [50] data suggest no differences in efficacy or toxicity for these sub-groups. Current trials are evaluating HF with reconstruction (Alliance221505/ NCT03414970; FABREC Trial/ NCT03422003). Furthermore, additional intensive regimens after lumpectomy (FAST: 28.5 Gy in 5 once-weekly fractions [51, 52]; FAST Forward: 26 Gy in 5 fractions over 1 week [53]) suggest early toxicity comparable to 40 Gy/15 fractions. These regimens may be considered in selected patients undergoing breast RT (without regional-nodal RT). Similarly, a boost should be reserved for patients with greatest absolute benefit (e.g., positive margins, age ≤ 40) [54].

Priority C patients are those in whom RT does not affect survival outcomes and includes all DCIS with exception of ER- DCIS with positive margins (Priority B3), if re-excision is not possible. Patients ≥ 65–70 years with early-stage, node negative, ER + invasive disease should start endocrine therapy after surgery, with RT safely omitted or deferred until the pandemic is over [55, 56]. A mechanism should be in place to re-evaluate patients for whom standard RT therapy is deferred so that recurrences/disease progression can be detected and managed appropriately (Table 4).

**Table 4:** Priority categories for radiation oncology

Priority	Patient Description	COVID-19 Treatment Considerations
Priority A		
A	Bleeding/painful inoperable local–regional disease, Symptomatic metastatic disease	Consider palliative HF regimens
A	Progression of disease during NAC	Consider definitive HF regimens
Priority B		
B1	Inflammatory BC s/p mastectomy	Consider PMRT HF regimens
B1	Node positive: TNBC or HER2 + disease s/p BCT or mastectomy	Consider WBRT or PMRT HF regimens
B1	Postmastectomy with 4 or more tumor-positive nodes	Consider PMRT HF regimens
B1	Residual node-positive disease after NAC	Consider WBRT or PMRT regimens
B2	PMRT with 1–3 tumor-positive nodes	Consider PMRT HF regimens
B2	Node negative: TNBC or HER2 + s/p BCT	Consider WBRT HF regimens
B2	If tumor-positive margin after BCT for invasive BC with no alternative therapy options*	Consider WBRT HF regimens
B3	If tumor-positive margin after BCT for invasive BC with alternative therapy options	Consider WBRT HF regimens
B3	Young age (≤ 40 years) s/p BCT, node negative with ≥ 1 additional high-risk features (LVI + , PNI +)	Consider HF regimens
B3	ER- DCIS with a positive margin	Consider HF WBRT regimens
Priority C		
C	DCIS^**^	Initiate endocrine therapy if ER + Defer radiation therapy until pandemic is over
C	> 65 years early-stage, nodenegative ER + / HER2- taking adjuvant endocrine therapy s/p BCT	Omit radiation therapy or defer until pandemic is over
Hypofractionated Regimens:		
Palliative Radiation		
4 Gy × 5 total 20 Gy		Meta-analysis [43]
8 Gy × 1 total 8 Gy		RTOG 97–14 [42]
Whole breast radiation therapy:		
2.67 Gy daily × 15 total 40.05 Gy		START B [45]
2.66 Gy daily × 16 total 42.56 Gy		Canadian [46]
5.7 Gy once per week × 5 total 28.5 Gy		FAST [52]
5.2–5.4 Gy daily × 5 total 26–27 Gy		FAST Forward [53]
Postmastectomy radiation therapy:		
2.5 Gy daily × 15 to chest wall total 37.50 Gy; 2.5 Gy daily × 14 to regional nodes (including IMN) total 35 Gy		British Columbia PMRT trial [49]
2.90 Gy × 15 daily to chest wall, SC & Level III axilla total 43.5 Gy No IMN or reconstruction		China PMRT Trial [50]
2.66 Gy daily × 16 to chest wall + regional nodes (with IMN) total 42.56 Gy		NCT03414970
2.67 Gy daily × 15 to chest wall total 40.05 Gy, 2.67 × 14 to RNI total 37.38 Gy		NCT03422003
Boost		
2.5 Gy × 4 total 10 Gy, consider additional 2.5 Gy fraction for positive margin		
Considerations for treatment interruptions		
No change to WBRT, PMRT dose. Adjust boost as follows: No boost in original treatment plan: Add boost 2.5 Gy × 4 Boost in original treatment plan: consider additional 2.5 Gy fraction to boost PTV total 12.5 Gy^***^		

*BC* breast cancer, *TNBC* triple negative breast cancer, *HF* hypofractionated, *NAC* neoadjuvant chemotherapy, *PMRT* postmastectomy radiation therapy, *WBRT* whole breast radiation therapy, *BCT* breast conserving therapy, *LVI* lymphovascular invasion, *PNI* peri-neural invasion, *ER* estrogen receptor, *HER2* human epidermal growth factor receptor 2, *IMN* internal mammary node; *RNI* regional-nodal irradiation

*TNBC with tumor positive margins should be given priority over TNBC with negative margins

**Exception to DCIS in Priority C is ER-negative DCIS with positive margin

***Adapted from Gay HA, et al. [57]

## Discussion

The three priority levels we have defined reflect the urgency of treatment during this pandemic resulting in multidisciplinary management recommendations taking into account the burden of the COVID-19 pandemic on our health system.

### Hospital factors to consider in prioritization of BC patients

It is critical for physicians to understand the rapidly changing local conditions and available resources as well as risks/benefits of various treatments for patients, staff, and hospital systems. Evolving local conditions and resources will influence which priority category receives treatment. Factors to consider include supply and equipment inventory (ventilators, PPE) and availability of intensive care and inpatient beds. Other factors would be the proportion of healthcare personnel infected with SARS CoV-2, and whether BC treatment will put healthcare workers at risk. Finally, the prevalence of regional community transmission will determine hospital capacity for outpatient care. These aforementioned factors will determine which BC patients receive treatment during or after the pandemic resolves.

### Importance of the multidisciplinary approach to the BC patient

The basic tenets of cancer care coordination should be followed as much as possible during the COVID-19 pandemic. Management of BC patients requires a highly integrated and multidisciplinary approach. An intervention in one specialty will have a direct impact on another specialty. For example, the American College of Surgeons issued a statement that elective surgeries should be canceled [17]. Fortunately, many BC patients do not need upfront surgical resection because of neoadjuvant treatment options. However, deferring BC cases will initially increase the medical oncologist workload and will result in a backlog of procedures when the pandemic resolves. For these reasons, multidisciplinary discussion documenting Priority category for surgery and/or adjuvant treatments is necessary to ensure the best outcomes for patients. If feasible, tumor board discussions should include both standard and COVID-19 recommendations based on institution’s level of pandemic severity. Documentation of these discussions in the medical record is highly recommended.

### Future directions

This information should be used to organize a process of structured decision-making for the care of patients with breast disease during the COVID-19 pandemic. However, as the pandemic rapidly evolves, we are increasingly learning about viral transmission and its impact on the health system; thus, these recommendations will evolve over time with continued updates. This consortium will continue to adapt these recommendations to the current pandemic severity including future waves of the COVID-19 pandemic. It is our hope that these current recommendations will help clinicians provide the highest quality care for their patients during this evolving pandemic.
